# Residual risk of hepatitis B virus transmission through blood donations in Burkina Faso screened with rapid diagnostic tests

**DOI:** 10.1002/hsr2.748

**Published:** 2022-08-08

**Authors:** Armel M. Sanou, Achille S. Nikièma, Seimbou Zalla, Mamadou Ouattara, Nina Pascaline S. Dakouo, Alice Kiba‐Koumare, Mariam Seynou, Delphine Napon‐Zongo, Roger Sombié

**Affiliations:** ^1^ Laboratoire de Microbiologie Clinique et d'Immunologie Institut de Recherche en Sciences de la Santé (IRSS) Bobo‐Dioulasso Burkina Faso; ^2^ Laboratoire de Parasitologie, Unité Paludisme et Maladies Tropicales Négligées Institut de Recherche en Sciences de la Santé (IRSS) Bobo‐Dioulasso Burkina Faso; ^3^ Direction de la qualité et des vigilances, Centre National de Transfusion Sanguine (CNTS) Avenue Kumda Yoonré Ouagadougou Burkina Faso; ^4^ Service Scientifique et Technique, Centre de Recherche en Santé de Nouna (CRSN) Institut National de Santé Publique (INSP) Nouna Burkina Faso; ^5^ Service de Médecine Interne, Endocrinologie et de Maladies Métaboliques Centre Hospitalier Universitaire de Bogodogo Ouagadougou Burkina Faso; ^6^ Unité de Formation et de Recherche en Sciences de la Santé (UFR/SDS) Université Joseph Ki‐Zerbo Ouagadougou Burkina Faso

**Keywords:** blood donation, Burkina Faso, HBV, HCV, HIV, RDTs

## Abstract

**Background and Aims:**

hepatitis B virus (HBV), hepatitis C virus (HCV), and human immunodeficiency virus (HIV) represent the major transfusion–transmissible pathogens worldwide. The risk of transmission is relatively high in African countries, mainly due to unreliable screening methods of blood donations. In Burkina Faso, predonation screening using rapid diagnostic tests (RDTs) is widespread, raising the major question of the transfusion safety in the country. The objective of this study was to assess the risk of transmission of HBV, HCV, and HIV through blood transfusion in the context of the use of RDTs for screening of the blood donations.

**Methods:**

In this cross‐sectional study, a total of 417 serum samples obtained from blood donors tested negative for HBsAg, anti‐HCV, and anti‐HIV using RDTs were retested for the same markers using chemiluminescent immunologic assays. Total antibodies to HBV core (anti‐HBc) were tested on randomly selected samples. HBV‐DNA and HCV‐RNA viral loads (VLs) were quantified on HBsAg and anti‐HCV positive samples, respectively. To assess possible occult hepatitis B infection (OBI), HBV‐DNA‐VL was quantified on 313 randomly selected HBsAg‐negative samples.

**Results:**

HBsAg and anti‐HCV were found respectively in 6 (6/417; 1.4%) and 11 (11/417; 2.6%) samples. No samples were reactive for anti‐HIV. Total anti‐HBc were detected in 217 out of the 319 randomly selected samples (217/319; 68.02%). HBV‐DNA was detected in four (4/313; 1.27%) samples, including two (2/6; 33.33%) of the six HBsAg positive samples and two (2/313; 0.6%) of the HBsAg‐negative samples, suggesting two cases of occult HBV infection. All anti‐HCV antibody‐positive samples were HCV‐RNA negative.

**Conclusion:**

This study shows that RDTs are not sufficiently sensitive for the screening of blood donations. Our results highlight the urgent need to think about the extension of sensitive immunological tests in all blood transfusion centers and also the implementation of nucleic acid amplification techniques.

## 1 INTRODUCTION

According to the World Health Organization (WHO), viral hepatitis remains a real concern worldwide, mainly in Sub‐Saharan Africa and South‐East Asia. Each year, about 1.34 million people die from hepatitis infections and 96% of these deaths are attributable to hepatitis B virus (HBV) and hepatitis C virus (HCV).^1^ With an estimated 36.9 million people living with human immunodeficiency virus (HIV) and more than 35 million deaths to date, HIV infection continues to be a major global public health problem.^2^ HBV, HCV, and HIV represent the major transfusion–transmissible pathogens worldwide. The risk of transmission of these pathogens varies geographically and it is relatively high in many African countries due to the lack of quality assurance and the use of unreliable detection methods like rapid diagnostic tests (RDTs) for the screening of blood donations.^3^, ^4^, ^5^ The performance of these tests is highly variable, but top tests ranked by the WHO comparative study allow effective screening of blood donors. However, top tests tend to be more expensive, which leads to the selection of less effective tests that are most often cheaper.

To address this situation, the WHO advocates the implementation of various strategies including mandatory screening of all blood donors for major transfusion–transmissible infections by high‐quality laboratory testing.^6^ In addition, WHO considers the implementation of transfusion safety as a key step towards the elimination of viral hepatitis as a health problem by 2030 in low‐ and middle‐income countries (LMICs).^1^

In Burkina Faso, with respective prevalence rates of 9.1%, 3.6%, and 0.8% in the general population, HBV, HCV, and HIV infections remain major public health problems.^7^, ^8^ Likewise, high prevalences of these pathogens were also reported among blood donors^9^, ^10^, ^11^, ^12^, ^13^ highlighting the risk of transmission through blood transfusion. Blood transfusion safety (BTS) in the country is effective since 2000 and is implemented by the National Blood Transfusion Center (NBTC). BTS relies upon the systematic screening of all blood products for HIV‐1/2, HBV, HCV, and syphilis infections using enzyme‐linked immunosorbent assay (EIA) and/or chemiluminescent immunologic assay (CLIA). With these assays, the residual transmission risk was evaluated to be 1/1366 for HIV, 1/408 for HBV, and 1/213 for HCV.^14^ However, because EIA and CLIA are not available everywhere, and centralized testing has shown its limitations, several health facilities implement BTS through rapid test‐based screenings. Although offering economic advantages for resource‐limited countries, the imperfect sensitivity of these tests, raise concerns about the safety of the blood transfusion system.^13^ Previous studies reported data on the performance of the rapid tests in blood donors suggesting a potential risk of posttransfusion infections.^15^, ^16^, ^17^, ^18^ To our knowledge, although widely used for the biological qualification of donations in Burkina Faso, no studies to date have evaluated the residual risk of pathogen transmission following the use of RDTs. Indeed, most of the used RDTs have neither been accredited nor evaluated beforehand for the screening of blood products. This study aimed to assess the risk of transmission of HBV, HCV, and HIV through blood donations in Burkina Faso screened with RDTs.

## 2 MATERIALS AND METHODS

### 2.1 Study design and setting

**Figure 1:**
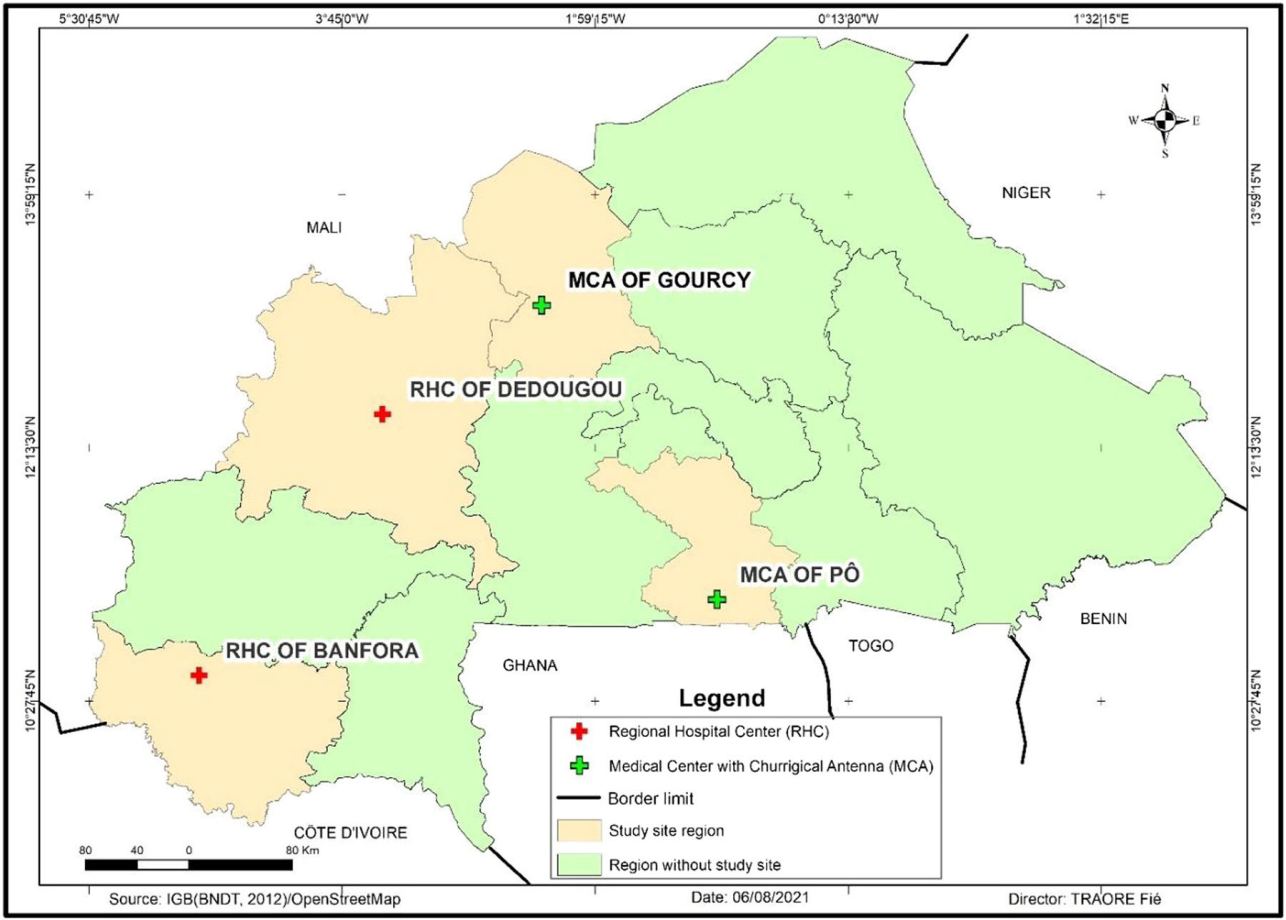
Map showing the localization of the study sites.

This cross‐sectional descriptive study was conducted between November 2019 and March 2020 in four health centers of Burkina Faso, the Regional Hospital (RH) of Banfora in the “Cascades” region, the RH of Dédougou in the “Boucle du Mouhoun” region, the Medical Center (MC) of Pô in the Centre‐South region and the MC of Gourcy in the North region (Figure 1). The RHs of Banfora and Dédougou are 90 and 180 km, respectively from the Bobo‐Dioulasso Regional Blood Transfusion Centers (RBTCs). As for the MCs of Pô and Gourcy, they are respectively 147.5 and 140.5 km from the RBTC of Ouagadougou. However, not being able to cover the entire national territory (NBTC), these health facilities are authorized and obliged to implement BTS through rapid test‐based screening. All of them have a blood bank department and were randomly selected from 39 health centers that use RDTs for the screening of blood.

### 2.2 Study population and data collection

We enrolled blood donors screened negative for hepatitis B surface antigen (HBsAg), HCV antibodies (anti‐HCV), and HIV‐1/2 antigen/antibodies (anti‐HIV) using RDTs, and who signed an informed consent form to participate in the study. Venous whole blood sample was collected from each participant into a dry tube. Sera were obtained after centrifugation of the samples at 4000 rpm for 5 min and were stored at −80°C until analysis. In addition, sociodemographic characteristics and knowledge about viral hepatitis were recorded using a structured questionnaire. These data included age, sex, education, occupation, marital status, viral hepatitis transmission routes, and immunization status. Detailed information about the RDTs used during the study period at each site are presented in Table 1.

**Table 1:** Characteristics of the RDTs used during the study period for predonation screening of HBsAg, anti‐HCV, and anti‐HIV‐1/2

Characteristics	HBsAg RDTs			Anti‐HCV RDTs			HIV‐1/2 RDT
	CHIL® HBsAg	SD Bioline® HBsAg	JusCheck_®_ Cassette Test Rapid HBsAg	CHIL® HCV cassette	SD Bioline® HCV	HCV Gold Rapid Screen Test Strip	Alere determine HIV‐1/2 Ag/Ab Combo test
Sites where tests were used	Pô	Banfora Dédougou	Gourcy	Pô	Banfora Dédougou	Gourcy	Banfora Dédougou Gourcy Pô
Laboratory	CHIL Medical Supplies Ltd., Turkey	Standard Diagnostics. South Korea	AllTest Biotech Co., Ltd., China	CHIL Medical Supplies Ltd., Turkey	Standard Diagnostics. South Korea	Span Biotech Ltd., China	Orgenics Ltd.
Reaction principle	IC	IC	IC	IC	IC	IC	IC
Specimen type	Plasma/serum	Plasma/serum	Whole blood/plasma/serum	Plasma/serum	Plasma/serum	Whole blood/plasma/serum	Whole blood/plasma/serum
Storage temperature (°C)	10–30	1–30	2–30	10–30	1–30	4–30	2–30
Sample volume	2–3 drops	100 µl	75 µl	2–3 drops	100 µl	70–75 µl	50 µl
Analytical threshold	2 ng/ml	3 ng/ml	1 ng/ml	–	–	–	12 pg/ml
Se according the manufacturer	98.84%	100%	>99.9%	98.84%	100%	>99.9%	HIV‐1/2: 100%
Sp according the manufacturer	98.94%	100%	99.5%	98.94%	100%	99.5%	99.75%
Unit cost (USD)	$ 0.73	$1.54	–	$0.73	–	$2.65	$1.16

Abbreviations: HCV, hepatitis C virus; HIV, human immunodeficiency virus; IC, immunochromatographic; RDT, rapid diagnostic test.

### 2.3 Laboratory analysis

All serum samples were tested for HBsAg, anti‐HCV, and anti‐HIV using the CLIA on the Cobas® 6000 analyzer (Elecsys® HBsAg II, Elecsys® Anti‐HCV II, Elecsys® HIV combi PT; Roche Diagnostics). Total anti‐HBc were tested on samples randomly selected using CMIA (Elecsys® Anti‐HBc II; Roche Diagnostics). HBV‐DNA and HCV‐RNA viral loads (VLs) were quantified respectively on HBsAg and anti‐HCV‐reactive samples using the Roche Molecular System (COBAS AmpliPrep/COBAS Taqman HBV Test Version 2.0; COBAS AmpliPrep/COBAS Taqman HCV Test Version 2.0). To detect possible occult hepatitis B infection, HBV‐DNA‐VL was quantified in HBsAg‐negative samples randomly selected (COBAS AmpliPrep/COBAS Taqman HBV Test Version 2.0). The manufacturer's reported limits of detection for HBsAg, HIV (p24 antigen), HBV DNA, and HCV RNA were 0.1 (0.88ng/ml), ≤2, 20, and 15 IU/ml, respectively. As for Elecsys® Anti‐HCV II, the sensitivity and specificity were 100% and 99.9%, respectively, and 100% for both for Elecsys® Anti‐HBc II. With respect to the cost of testing, serological markers have been estimated to cost approximately $7.00 per test, and approximately $25.00 for molecular testing. All tests were performed according to the manufacturer's instructions.

### 2.4 Statistical analysis

Statistical analyses were performed using STATA SE version 14.0 software. We used proportions to describe categorical variables and means (standard deviation) to describe continuous variables. Risk of transmission was computed for each pathogen considering CLIA as a gold standard. Statistical inferences were based on 95% confidence intervals (CIs).

### 2.5 Ethical considerations

This study received the approval of the Ethics Committee of the “Institut de Recherche en Sciences de la Santé” before its implementation (17‐2019/CEIRS‐20062019). All participants and legally authorized representatives of illiterate participants provided written informed consent and donors who were tested positive for one or several markers, were referred to a Health Center. The research was performed in accordance with the Declaration of Helsinki.

## 3 RESULTS

### 3.1 Sociodemographic characteristics of participants and their knowledge about hepatitis viruses

During the study period, a total of 2994 blood donors were tested, and 57 (1.9%) were found positive for HIV, 252 (8.4%) for HBV, 90 for anti‐HCV (3.0%), and 46 (1.5%) for syphilis. Of the blood donors screened negative for these markers (2549), 417 from Banfora (*n* = 122/417; 29.2%), Dédougou (*n* = 116/417; 27.8%), Pô (*n* = 125/417; 29.9%), and Gourcy (*n* = 57/417; 12.9%) were enrolled. The mean age of the donors was 33.45 years (age range: 18–57 years), 297 (297/417; 71.2%) were male and 211 (211/417; 50.6%) were students. The age group from 18 to 29 years was the most represented with 303 (303/417; 72.6%) participants. Among the donors, 214 (214/417; 51.3%) had heard of viral hepatitis and 102(102/417; 24.5%) had knowledge of transmission routes. As for their hepatitis B immunization status, only 14 (14/417; 3.4%) of the participants reported being fully vaccinated. The detailed characteristics of the study population are summarized in Table 2.

**Table 2:** Sociodemographic characteristics and knowledge about viral hepatitis of blood donors

Characteristic	Total number (*N* = 417)	Percentage (%)
Sex		
Male	297	71.22
Female	120	28.78
Age (years)		
(18–20)	107	25.65
(20–30)	196	47.00
(30–40)	71	17.03
40+	43	10.31
Level of education		
None	70	16.79
Elementary	46	11.03
Secondary/higher	301	72.18
Occupation		
Employee	42	10.07
Merchant	67	16.07
Farmer	66	15.83
Student (secondary high schools/universities)	211	50.60
Others	31	7.43
Marital status		
Single	268	64.27
Married	149	35.73
Heard about viral hepatitis		
No	203	48.68
Yes	214	51.32
Knowledge of transmission route		
No	315	75.54
Yes	102	24.46
Immunization status		
Not vaccinated	216	51.80
Don't know	187	44.84
Fully vaccinated	14	3.36

### 3.2 Risk of transmission

Using the CLIA method, HBsAg and anti‐HCV were detected respectively in 6 (1.4%; 95% CI: 0.5–3.1) and 11 (2.6%; 95% CI: 1.3–4.7). No samples were reactive for HIV‐1/2 infection. Of the 319 randomly selected samples, including the six HBsAg‐reactive samples, total anti‐HBc was detected in 217 (68.02%; 95% CI: 62.2–72.7). HBV‐DNA was detected in four (1.275%; 95% CI: 0.03, 2.94) samples including two (2/6; 33.33%) in the sikx reactive HBsAg samples and two (2/313; 0.6%) in the nonreactive HBsAg samples, this latter result arguing for two cases of occult hepatitis B. In HBsAg‐reactive samples, HBV‐DNA‐VL were 16.400 and 932 IU/ml, and for occult hepatitis B infection (OBI) cases, they were 21 and 38 IU/ml. All HBV‐DNA detectable samples were reactive for anti‐HBc. HCV‐RNA was not detected in any of the anti‐HCV‐reactive samples. The serological and molecular results are summarized in Table 3.

**Table 3:** Serological and molecular results

Markers	*N*	%	95% CI
HBsAg			
Nonreactive	411	98.56	
Reactive	6	1.44	(0.52, 3.10)
Anti‐HCV			
Nonreactive	406	97.36	
Reactive	11	2.64	(1.32, 4.67)
anti‐HBc (*n* = 319)			
Nonreactive	102	31.98	
Reactive	217	68.02	(62.16, 72.72)
HBV DNA (IU/ml) (*n* = 313)			
Undetectable	309	98.72	
Detectable	4	1.27	(0.03, 2.94)

Abbreviations: CI, confidence interval; HBV, hepatitis B virus;  HCV, hepatitis C virus.

A multivariable logistic regression analysis reported a statistically significant correlation between knowledge of viral hepatitis (transmission routes) and HBsAg seronegativity (odds ratio [OR] = 6.38; 95% CI: 1.15–35.40; Table 4). However, no significant association between anti‐HCV and anti‐HBc positivity and participants' demographics was found.

**Table 4:** Factor associated with HBsAg and anti‐HCV seroreactivity using CLIA

		HBsAg			Anti‐HCV		
Variable	*N*	*n* (%)	OR (95% CI)	*p*	*n* (%)	OR (95% CI)	*p value*
Age group				0.4923			0.789
(18–20)	107	3 (2.86%)	1		3 (2.80%)	1	
(20–30)	196	2 (1.02%)	0.35 (0.06, 2.13)		5 (2.55%)	0.89 (0.20, 3.80)	
(30–40)	71	0 (0.00%)	1		3 (4.23%)	1.5 (0.29, 7.65)	
40+	43	1 (2.33%)	0.81 (0.08, 8.01)		0 (0.00%)		
Gender				0.8066			0.1047
Male	297	4 (1.35%)	1		10 (3.37%)	1	
Female	120	2 (1.67%)	1.24 (0.22, 6.87)		1 (0.83%)	0.24 (0.03, 1.90)	
Education							0.9527
None	70	0 (0.00%)			2 (2.86%)	1	
Elementary	46	0 (0.00%)			0 (0.00%)		
Secondary/higher	301	6 (1.99%)			9 (2.99%)	1.04 (0.22, 4.96)	
Occupation				0.9965			0.9297
Employee	42	1 (2.38%)			0 (0.00%)		
Merchant	67	0 (0.00%)			2 (2.99%)	1	
Farmer	66	0 (0.00%)			2 (3.03%)	0.9 (0.18, 4,42)	
Student	211	5 (2.37%)	0.99 (0.11, 8.74)		7 (3.32%)	0.91 (0.18, 4.49)	
Other	31	0 (0.00%)			0 (0.00%)		
Marital status				0.2973			0.5448
Single	268	5 (1.87%)			8 (2.99%)	1	
Married	149	1 (0.67%)	0.35 (0.04, 3.07)		3 (2.01%)	0.66 (0.17, 2.56)	
Heard about viral hepatitis				0.0979			0.8281
No	203	1 (0.49%)			5 (2.46%)	1	
Yes	214	5 (2.34%)	4.83 (0.55, 41.72)		6 (2.80%)	1.14 (0.34, 3.80)	
Knowledge of transmission routes				0.0279			0.1843
No	315	2 (0.63%)			10 (3.17%)	1	
Yes	102	4 (3.92%)	6.38 (1.15, 35.40)		1 (0.98%)	0.3 (0.04, 2.89)	
Immunization status				0.1215			0.2444
Not vaccinated	216	5 (2.31%)	1		4 (1.85%)	1	
Don't know	187	1 (0.53%)	0.22 (0.03, 1.96)		7 (3.74%)	2.06 (0.59, 7.15)	
Fully vaccinated	14	0 (0.00%)	1		0 (0.00%)		

Abbreviations: CI, confidence interval; CLIA, chemiluminescent immunologic assay;  HCV, hepatitis C virus; OR, odds ratio.

## 4 DISCUSSION

In Burkina Faso, BTS implemented by the NBTC consists of systematic screening for HIV, HBV, HCV, and syphilis using sensitive methods including EIA and/or CLIA techniques. However, two previous studies conducted in Blood Banks in Burkina Faso pointed out a significant residual risk of about 0.3%, 0.5%, and 0. 07% for HBV, HCV, and HIV, respectively.^14^, ^19^ To increase the population's access to blood products, several health centers are authorized to practice blood transfusion after screening their donations with RDTs. We evaluated the risk of posttransfusion transmission of these major pathogens and found 6 (1.4%) HBsAg‐reactive and 11 (2.6%) anti‐HCV‐reactive samples were missed by RDTs. These findings are in line with studies reporting low sensitivity of RDTs evaluated in African blood donors.^18^, ^20^ However, the 2.6% anti‐HCV detected in RDT‐negative samples appears high. This could be related to evidence that CLIA results were likely false positive. The nondetection of HCV RNA on all anti‐HCV‐reactive samples could be a confirmation.

No case of HIV infection was detected, suggesting the good quality of the *Alere Determine HIV‐1/2 Ag/Ab Combo test*, which has been approved by the national regulation structure (Agence Nationale de la Régulation Pharmaceutique) in Burkina Faso and also has WHO prequalification. In addition, a national screening algorithm for HIV using this test is available and is evaluated regularly contributing to reduce the risk of false results. Another explanation could be the low HIV prevalence estimated to 0.8% in the general population.^8^

Except for SD Bioline® tests, all tests used for HBV and HCV predonation screening have not been evaluated and approved in Burkina Faso, and do not have WHO prequalification. Consequently, information on their diagnostic performance on local samples are lacking and there is a risk of missing positive cases with these tests. The routine use of not registered tests in the country could be explained by the fact that the ordering of tests is mostly managed by nonspecialists in the field. Another reason may be the level of corruption in the health sector.^21^ This situation highlights the urgency of setting up a national system for evaluating tests before they are used in blood transfusion.

An overall prevalence of 68.02% of total anti‐HBc was found, indicating an important circulation of HBV in Burkina Faso.^7^, ^22^ Therefore, we wondered whether in this high proportion of anti‐HBc‐positive blood donors, OBI may be a matter of concern. Indeed, OBI represents a potential threat for BTS mainly in LMICs where the use of nucleic acid amplification techniques (NAAT) is not widespread.^23^ OBI is defined as the presence of replication‐competent HBV DNA in the liver and/or in the blood of HBsAg‐negative people. OBI can be categorized either as seropositive or seronegative for anti‐HBc and anti‐HBs antibodies.^24^ The rate of detection of HBV‐DNA in HBsAg‐negative/anti‐HBc positive samples is estimated to be 1.6%–38%.^25^ In our study, occult HBV infection OBI cases were detected in two (0.6%) blood donors with HBV‐DNA VL of 21 and 38 IU/ml. This prevalence is close to that reported by Fopa et al.^23^ in Cameroon (0.56%), but lower than that observed by Diarra et al.^26^ in Burkina Faso (7.3%), Olotu et al.^27^ in Nigeria (5.4%) and Said et al.^25^ in Egypt (17.2%). These differences could be explained by the fact that HBV‐DNA was screened in both anti‐HBc negative and positive samples in our study. Other factors such as the studied population and the techniques used for HBV‐DNA detection may be involved. The high total anti‐HBc prevalence was also predictable as all participants were born before the implementation of the hepatitis B vaccine in the national immunization program in 2006. Indeed, only 3.36% of the participants were fully vaccinated (Table 2).

Our results showed that HBV DNA was not detected on four HBsAg‐reactive samples. This could be due to the very low level of viral DNA that could not be detected by the COBAS Version 2.0 used in this study (analytical sensitivity: 20 IU/ml) for molecular analysis. In addition, since a confirmatory test for HBsAg was not used, false positive results cannot be excluded.

Based on these overall results, the implementation of sensitive methods in blood centers in Burkina Faso should be strongly considered. CLIA tests, because of their operating cost and capacity, are not suitable for small blood banks. However, they could be used in regional centers, and made accessible to peripheral blood banks through a centralized testing system. High‐performance RDTs could therefore significantly improve patient blood safety. To date, NAAT tests are available in almost all health centers in Burkina because of the response to the COVID‐19 pandemic. Thus, the detection of OBI cases by applying NAAT to pooled samples could be an interesting option since studies have reported transfusion transmission of HBV with blood components from donors with OBI.^28^

In our study, assessment of participants' knowledge about viral hepatitis revealed that half of the donors (48.68%) had never heard of viral hepatitis and 75.54% had no information on transmission routes, confirming data we reported earlier.^13^ Interestingly, a positive correlation (OR: 6.38; 95% CI: 1.15, 35.40) was found between the knowledge of the transmission routes and HBV seronegativity in the participants of this study (Table 4). Communication, information, and education activities must be organized for potential blood donors to raise awareness about this scourge. In addition, since students represented the main population of blood donors in our study (50.6%), awareness modules could be integrated into their curriculum.

Taking into account the objectives, the major limitation of our study was the relatively small sample size, which did not allow having a comprehensive picture of the magnitude of the transmission risk. Large‐scale studies are therefore needed. Another limitation is the absence of confirmation step HBsAg, anti‐HCV, and anti‐HIV assays.

In conclusion, our study showed a potential risk of transmission of HBV (1.44%) when using RDTs for qualification of blood donations. In addition, although HCV RNA was not detected in any samples confirming HCV infection, 11 anti‐HCV‐reactive samples were missed by the rapid tests. These results highlight the need to implement sensitives techniques in blood transfusion in Burkina Faso. OBI cases were also diagnosed in at least 0.6% of the donors. As Burkina Faso is a country of high endemicity for HBV, further confirmed here by high anti‐HBc positivity in our study cohort (about 68%), the implementation of NAAT in blood transfusion centers for the detection of OBI cases, while cost‐effective, should be definitely considered. This will constitute the main point of a high quality of our blood transfusion system.

## AUTHOR CONTRIBUTIONS

**Armel M. Sanou**: Conceptualization; data curation; formal analysis; funding acquisition; investigation; methodology; writing – original draft; writing – review and editing. **Achille S. Nikiéma**: Conceptualization; data curation; investigation; methodology; writing – review and editing. **Seimbou Zalla**: Conceptualization; data curation; investigation; methodology; writing – review and editing. **Mamadou Ouattara**: Methodology; formal analysis; software; writing – review and editing. **Mariam Seynou**: Methodology; formal analysis; software; writing – review and editing. **Alice Kiba‐Koumare**: Supervision; writing – review and editing. **Delphine Napon‐Zongo**: Supervision; writing – review and editing. **Roger Sombié**: Supervision; writing – review and editing.

## CONFLICT OF INTEREST

The authors declare no conflict of interest.

## TRANSPARENCY STATEMENT

Armel M. Sanou affirms that this manuscript is an honest, accurate, and transparent account of the study being reported; that no important aspects of the study have been omitted; and that any discrepancies from the study as planned (and, if relevant, registered) have been explained. All authors have read and approved the final version of the manuscript. Armel M. Sanou had full access to all of the data in this study and takes complete responsibility for the integrity of the data and the accuracy of the data analysis.

## Data Availability

All data generated or analyzed during this study are included in this article and its Supporting Information files data and can be requested from the corresponding author.
